# Assessing the impact of state “opt-out” policy on access to and costs of surgeries and other procedures requiring anesthesia services

**DOI:** 10.1186/s13561-017-0146-6

**Published:** 2017-02-28

**Authors:** John E. Schneider, Robert Ohsfeldt, Pengxiang Li, Thomas R. Miller, Cara Scheibling

**Affiliations:** 1 Avalon Health Economics, LLC, 26 Washington Street, 3rd Fl, 07960 Morristown, NJ USA; 2 Health Policy & Management, Texas A&M University, 212 Adriance Lab Rd, 1266 TAMU, 77843-1266 College Station, TX USA; 3 Division of General Internal Medicine, University of Pennsylvania, 423 Guardian Dr, Philadelphia, PA 19104 USA; 4 American Society of Anesthesiologists, 1061 American Lane, 60173-4973 Schaumburg, IL USA; 5 Avalon Health Economics, 26 Washington Street, 3rd Fl., 07960 Morristown, NJ USA

## Abstract

In 2001, the U.S. government released a rule that allowed states to “opt-out” of the federal requirement that a physician supervise the administration of anesthesia by a nurse anesthetist. To date, 17 states have opted out. The majority of the opt-out states cited increased access to anesthesia care as the primary rationale for their decision. In this study, we assess the impact of state opt-out policy on access to and costs of surgeries and other procedures requiring anesthesia services. Our null hypothesis is that opt-out rule adoption had little or no effect on surgery access or costs. We estimate an inpatient model of surgeries and costs and an outpatient model of surgeries. Each model uses data from multiple years of U.S. inpatient hospital discharges and outpatient surgeries. For inpatient cost models, the coefficient of the opt-out variable was consistently positive and also statistically significant in most model specifications. In terms of access to inpatient surgical care, the opt-out rules did not increase or decrease access in opt-out states. The results for the outpatient access models are less consistent, with some model specifications indicating a reduction in access associated with opt-out status, while other model specifications suggesting no discernable change in access. Given the sensitivity of model findings to changes in model specification, the results do not provide support for the belief that opt-out policy improves access to outpatient surgical care, and may even reduce access to outpatient surgical care (among freestanding facilities).

## Background

In 2001, the U.S. federal government released a rule that allowed states to “opt-out” of the federal requirement that a physician supervise the administration of anesthesia by a nurse anesthetist. The “November 13” rule was effective upon publication in the November 13, 2001 *Federal Register*. [1] For a state to opt-out of the federal supervision requirement, the state's governor must send a letter of attestation to the Centers for Medicare and Medicaid Services [1]. The letter must attest that: 1) the state's governor has consulted with the state's boards of medicine and nursing about issues related to access to and the quality of anesthesia services in the state; 2) it is in the best interests of the state's citizens to opt-out of the current federal physician supervision requirement; and 3) the opt-out is consistent with state law.

To date, as shown in Appendix Table 6, 17 states have opted out. [2] The majority of the opt-out states cited increased access to anesthesia care as the primary rationale for their decision. [2] Collectively, in 2015 these states had about 73 million residents, or about 23% of the total resident population of the United States. [3] The majority of the opt-out states were sparsely populated states (e.g., Iowa, North Dakota, and Montana), with the notable exception of California, which nonetheless includes large rural areas interior to the heavily populated Pacific coast.

Following the implementation of the November 13 rule, the U.S. Agency for Healthcare Research and Quality (AHRQ) was charged with assessing whether anesthesia outcomes differed between opt-out states and other states. The study analyzed Medicare data for 1999 through 2005, and found no evidence that opting out of the oversight requirement resulted in increased inpatient deaths or complications. [4] Similarly, a recent Cochrane review concluded there was insufficient evidence to conclude whether quality of anesthesia care differed across nurse and physician anesthesiologists [5]. However, among the stated goals of the opt-out rule was to improve access to anesthesia care and control growth in its costs. [6] At the time of the rule, there was a potential shortage of anesthesiologists, at least in some regions and states. [7] The presumption was that allowing nurse anesthetist to practice without physician supervision would alleviate these shortages and thus enhance access to anesthesia care. The lower professional service costs for nurse anesthetist practicing without physician supervision also was presumed to lower anesthesia care costs.

Despite the importance of the presumed cost and access benefits of the opt-out rule, to date few studies have attempted to quantify changes in access and costs attributable to the opt-out rules. Sun et al. [8] utilize data from the National Inpatient Sample (NIS) to assess whether opt-out was associated with an increase in the percentage of patients receiving a therapeutic procedure among patients admitted for appendicitis, bowel obstruction, choledocholithiasis, or hip fracture. In a similar vein, using claims data for Medicare fee-for-service enrollees, Sun et al. [9] examine differences in average anesthesia utilization rates three years before and after out-out for opt-out states grouped by year of opt-out, compared to differences in average anesthesia utilization rates over the same time period in non-opt-out states. Both studies conclude the adoption of the opt-out rule had no significant impact on access to anesthesia care.

In this study, we extend the literature on the impact of state opt-out policy by adding an assessment of its impact on costs of surgeries, and by assessing its impact on a wider variety of procedures requiring anesthesia services than in prior studies. Our hypothesis is that opt-out states exhibited changes in access to surgery and changes in surgery costs similar to non-opt-out states; that is, that the opt-out laws had little or no effect on surgery access or costs. We estimate models of inpatient surgery costs and surgery volume, as well as a model for volume of outpatient surgeries. Each model uses data from multiple years of U.S. inpatient hospital discharges and outpatient surgeries. Our results indicate that the opt-out policy is associated with higher inpatient surgery costs, with little or no impact on access for either inpatient or outpatient surgery.

## Methods

We used two data sources that were appropriate for the study objectives. There has been continuous growth in outpatient surgery both in years before and years after passage of the opt-out law. [9] Thus, we believe that it is important to examine access and cost associated with inpatient *and* outpatient surgery. We used the Nationwide Inpatient Sample (NIS) for analysis of changes in inpatient surgery volume. The NIS is part of the Healthcare Cost and Utilization Project (HCUP), and is the largest publicly available all-payer inpatient health care database in the United States, yielding national estimates of hospital inpatient stays (https://www.hcup-us.ahrq.gov/nisoverview.jsp#data). Unweighted, the NIS contains data from more than 7 million hospital stays each year. Weighted, it estimates (or represents) more than 36 million hospitalizations nationally (around 20%). With more than 20 years of data, the NIS is ideal for longitudinal analyses.

However, the database has undergone changes over time, including the sampling and weighting strategy used. Beginning in 2012, sampling strategy for NIS was redesigned from formerly a random sample of hospitals and retaining all discharges from those sampled hospitals to a random sample of discharges from all hospitals participating in HCUP. To remove inconsistency due to change of sampling strategy, we did not include NIS data for hospitalizations after 2011. Thus, our NIS sample covers a 14-year time frame from 1998 to 2011 which allows for several years before and after the opt-out decisions by states. The unit of observation is “facility-year.”

For outpatient surgery, we used the State Ambulatory Surgery and Services Databases (SASD). The SASD is also part of the HCUP system (https://www.hcup-us.ahrq.gov/sasdoverview.jsp). The SASD include encounter-level data for ambulatory surgeries and “may also include various types of outpatient services such as observation stays, lithotripsy, radiation therapy, imaging, chemotherapy, and labor and delivery.” The specific types of ambulatory surgery and outpatient services included in the SASD vary by state and data year. SASD include data from hospital-owned ambulatory surgery facilities and nonhospital-owned facilities.

For the outpatient analysis, we included three opt-out states (California, Colorado, and Kentucky) and three non-opt-out states (Florida, Maryland, and New Jersey). These states were selected based on two criteria: [1] the state-level SASD contain all of the data we will need to estimate the models (e.g., procedure codes); and [2] the state SASD data contain the sufficient pre- and post-opt-out years. The unit of observation for the outpatient analysis is also the “facility-year.”

Our outcomes include measures of access and cost. The access measures were the number of all inpatient and outpatient surgeries.^1^ The cost measure was average cost per surgical inpatient stay, calculated by using hospital cost-to-charge ratios to deflate total charges per stay reported in the NIS. Nominal cost estimates were converted to constant 2011 dollars using the “Hospital and related services” component of the Consumer Price Index (http://www.bls.gov/cpi/). No cost-to-charge ratio estimates are available for the outpatient facilities in the SASD, and as a result, no average cost estimates are available for outpatient procedures.

A quasi-experimental study design was used to study the change in outcomes (access and costs) in “treatment” facilities (those located in opt-out states) before and after opt-out policy implementation, compared to facilities located in non-opt-out states over the same time period. The statistical analysis was based on panel data facility-level fixed-effect model which examined how the change of opt-out status affected changes in outcomes while removing facility-level time-invariant unmeasured confounders. We used robust standard error estimation adjusting for state level clustering. The null hypothesis is that opt-out states exhibited changes in access to surgery and changes in surgery costs similar to non-opt-out states; that is, that the opt-out laws had little or no effect on surgery access or costs.

The base statistical model of access is written as:

$$ {\mathrm{D}}_{\mathrm{i}\mathrm{t}}=\alpha +{\upbeta}_1{\mathrm{OPT}}_{\mathrm{i}\mathrm{t}}+{\upbeta}_{\mathrm{n}}{\mathrm{X}}_{\mathrm{i}\mathrm{t}}+{\upbeta}_{\mathrm{n}}{\mathrm{T}}_{\mathrm{t}}+{\mathrm{U}}_{\mathrm{i}}+{\upvarepsilon}_{\mathrm{i}\mathrm{t}} $$

The unit of observation in the NIS is the discharge, and in the SASD is the procedure. In this equation, the dependent variable D_it_ refers to access (total number of surgeries) or cost (mean cost per surgery) for facility *i* in year *t*. The key right-hand side variable of interest is a dummy variable OPT_it_ indicating whether the facility is located in an opt-out state (OPT equal to 1 if the facility was located in an opt-out state and 0 otherwise) in year t (For example, CA adopted opt-out in 2009; thus OPT_it_ = 0 before 2009 and OPT_it_ = 1 since 2009 for CA). For a control state like FL, OPT_it_ = 0 during all the observed years [see Appendix Table 6]). X_it_ represents a vector of covariates likely to affect access or cost.

In the inpatient models, X_it_ includes facility characteristics (bed size^2^ of hospital: [1] small, [2] medium, [3] large; control/ownership of hospital: (0) government or private, collapsed category, [1] government, nonfederal, public, [2] private, non-profit, voluntary, [3] private, invest-own, [4] private, collapsed category; rural or urban hospital; and teaching or non-teaching hospital).^3^ The inpatient models also adjust for lagged (year t-1) facility-level patient summary measures, including the total number of hospitalizations, patient case mix (i.e. percentage of cases were female, mean length of stay, percentage of surgical cases, the mean of the Centers for Medicare & Medicaid Services Hierarchical Condition Category (CMS-HCC) risk score [9], age distribution [<18, 18 to 44, 45 to 64, 65 to 74, and 75 or older]), admission type [elective, emergency, or other], percentage of routine discharge hospitalizations, health insurance type [Medicare, Medicaid, private insurance, or others], and race [white, black, Hispanic, or others]). CMS-HCC risk adjustment was developed by CMS to produce a health-based measure of future medical need which has shown to be a significant predictor of medical costs and has a better predictive accuracy on mortality than the Charlson and Elixhauser methods [10]. A Herfindahl-Hirschman Index (HHI), with the market definition based on area patient flows,^4^ was used to adjust for area hospital market concentration. County-level variables potentially affecting access or cost also were included (i.e. total number of residents in the county, percentage of the population in poverty, percentage of the population who are Medicare beneficiaries, percentage of people between age 16 and 64, the unemployment rate, per capita income, and the number of anesthesiologists [MD/DO] per 10,000 residents).^5^ The remaining variables are dummy variables for time (T). Ui is facility-level time-invariant unmeasured variable. The error term is indicated as ε_it_.

Many of the variables available in the NIS included in the inpatient models were not available in the SASD. In the multiple regression models focusing on outpatient surgery, we used all model covariates available in the SASD. The data do not allow identification of the county location of freestanding outpatient facilities. Thus, the outpatient models focusing on the sample of all outpatient facilities account for lagged (year t-1) factors (patient flow, risk score, disposition status, and payment source variables), and a dummy variable for freestanding outpatient facilities (vs. hospital outpatient surgery departments). We addressed the differences (and changes) in access in rural versus urban areas by including an interaction terms of urban/rural indicator and opt-out indicator in the multiple regression models. Alternative models examine dependent variables measured in natural units and log transformations.

We conducted extensive sensitivity analysis to check the robustness of our findings. First, for the NIS, we examined using alternative definitions of access: 1) Removing cases age less than 18 out of total surgical discharges; 2) Removing all transplant Diagnosis-Related Groups (DRGs) and any craniotomy DRGs; and 3) limiting discharges to only hip and knee surgery procedures (DRG 209, 471, 503, 544, 471, or 545) and mean cost per discharge based on the definition. Because many pediatric procedures are performed in children’s hospitals where anesthesiologists provide solo care or are part of care team, and given that children are a unique population (with parents making health care decisions), the impact opt-out may be different from the impact on the adult population. Likewise, transplants and craniotomy represent very complex cases where, given current practice patterns, a low percentage of nurse anesthetists would be able to practice without physician supervision for those procedures. Hips and knees were examined separately because they represent a group of very common and fast growing procedures which are often performed in community hospitals.

Second, we examined robustness of our finding by varying covariates included in the models. In the SASD, we estimate separate models by freestanding status, a model focusing on the volume of specific outpatient procedures likely to require general anesthesia, and a model excluding the lagged patient flow variables. To examine whether early opt out have a different impact on outcomes compared to late opt out states, we conducted a set of sensitivity analyses in NIS sample. We repeated the analysis among early opt out states [states with opt out between 2001 and 2005 (i.e., IA, MN, NE, NH, NM, AK, KS, ND, OR, WA, MT, SD, WI) compared with non-opt out states during the period, and late opt out states (states with opt out between 2009 and 2011 (i.e., CA and CO) compared with non-opt out states during the period; in the whole NIS sample, we also ran another model by including opt-out variable (equal 1 after the opt out states opt out) and late opt-out indicator (equal to 1 for CA and CO during the whole study period, 1998 to 2011; equal to zero for other states)]. The coefficients of interaction terms show the differentiated impact of opt-out for late opt-out states comparing to early opt-out states.

## Results

The final analytic files included 13,573 facility-year observations in the NIS sample and 9,994 facility-year observations in the SASD sample. Descriptive data for the main outcomes associated with the inpatient file (NIS) and outpatient file(SASD) are shown in Appendix Tables 7 and 8. The results for the inpatient cost models are shown in Table 1. When cost per discharge was the dependent variable, the estimated coefficient of the opt-out variable was positive and statistically significant (*p* < 0.01). The point estimate indicates that the cost per discharge was $1,815 higher in opt-out states relative to non-opt-out states. Similarly, in the log cost models, the estimated coefficient of the opt-out variable was positive and statistically significant. The point estimate indicates that the cost per discharge was about 8.7% higher in opt-out states relative to non-opt-out states.^6^

**Table 1:** Inpatient Cost Models, Linear and Log Linear

	Mean costs per surgical case		Log Mean costs per surgical case	
	b	t	b	t
Opt out	1815.33***	3.76	0.08*	2.43
Rural hospital	−584.32	−0.51	0.01	0.19
Hospital bed size				
Small (reference)				
Medium	85.99	0.13	−0.03	−0.68
Large	−1037.20	−1.45	−0.10	−1.83
Control/ownership of hospital				
Government or private, collapsed category (reference)				
Government, nonfederal, public,	1403.23	0.99	−0.04	−0.93
Private, non-profit, voluntary	1448.21	0.99	−0.15**	−2.85
Private, invest-own	1770.03	1.11	−0.01	−0.19
Private, collapsed category	3400.20	1.96	0.01	0.17
Teaching hospital	1648.59	1.22	−0.04	−1.30
Hospital HHI based on patient flow	11802.96	1.73	−0.26	−0.56
Lagged (year t-1) facility-level patient summary measures				
Total number of hospitalizations	−0.05	−0.54	−0.00	−0.44
Percentage of cases were female	−2308.72	−0.24	−0.71	−0.93
Mean length of stay	426.60	1.21	0.03	1.11
Percentage of surgical cases	14348.88	1.75	0.54	1.33
Mean (CMS-HCC) risk score	−438.02	−0.16	−0.27	−1.16
Age distribution (%)				
<18	7003.33	0.63	0.96	0.86
18_44 (reference)				
45_64	11426.77	0.91	0.18	0.20
65_74	9031.06	0.47	1.42	1.13
75 or older	8585.06	0.56	0.91	0.86
Admission type (%)				
Elective (reference)				
Emergency	−993.71	−0.50	−0.08	−0.52
Other	1733.18	0.84	0.20	1.85
Percentage of routine discharge hospitalizations	−4511.40	−0.85	−0.47	−1.35
Health insurance type (%)				
Private insurance (reference)				
Medicare	−2590.06	−0.56	−0.15	−0.42
Medicaid	−289.20	−0.06	−0.02	−0.09
Others	559.90	0.21	0.07	0.35
Race (%)				
White (reference)				
Black	5323.13	0.76	−0.11	−0.24
Hispanic	−7994.42	−1.27	−0.38	−1.04
Other	−2340.99	−1.35	−0.19	−1.63
County-level variable				
Total number of residents in the county	0.00	0.77	0.00	0.68
Percentage of people in poverty	42.77	0.38	0.01	0.75
Percentage of people are Medicare beneficiaries	−12526.56	−0.50	−2.32	−1.27
Percentage of people between age 16 to 64	−7064.95	−0.77	−0.31	−0.65
Unemployment rate	1553.06	0.12	−0.73	−0.93
Per capita income	0.10	1.06	0.00	1.63
Number of anesthesiologists [MD/DO] per 10,000 residents	−784.20	−1.39	−0.06	−1.41
Year dummy variables				
2001.year (reference)				
2002.year	466.64	1.66	0.14***	6.16
2003.year	1176.08**	3.62	0.26***	9.19
2004.year	1993.96***	4.18	0.36***	11.54
2005.year	2942.80***	5.06	0.48***	9.88
2006.year	4007.31***	5.60	0.57***	9.93
2007.year	5322.09***	5.76	0.69***	9.80
2008.year	6344.88***	5.84	0.79***	10.12
2009.year	7524.34***	6.37	0.92***	10.61
2010.year	9554.10***	6.17	1.06***	9.39
2011.year	10332.97***	5.79	1.13***	9.21
Constant	−2772.51	−0.16	9.06***	6.87
N	1,339		1,339	
R-squared (within)	0.7226		0.7946	

*Notes*: [1] t-statistics in parentheses; *p < 0.05; **p < 0.01; ***p < 0.001; [2] Some hospital-year do not have cost-to-charge ratios; therefore, cost measure was not available; [3] interaction term between opt-out and rural hospital status was not statistically significant; therefore, main models do not include interaction terms; [4] Costs were in 2011 dollar adjusted by hospital and related services CPI

For the inpatient access models (Table 2), the opt-out variable coefficient was positive but not statistically significant in the model with the number of hospital discharges as the dependent variable. The magnitude of the point estimate implies an increase in surgical discharges that is small in magnitude – about 40 annually, or about 1.8% (based on the sample mean). Similarly, in the model that used the log of discharges as the dependent variable, the estimated coefficient of the opt-out variable is positive but not statistically significant.

**Table 2:** Inpatient Access Models, Linear and Log Linear

	Total number of surgical discharges		Log Total number of surgical discharges	
	b	t	b	t
Opt out	39.78	0.62	0.05	1.08
Rural hospital	−78.00	−0.87	0.05	0.35
Hospital bed size				
Small (reference)				.
Medium	20.62	0.77	−0.01	−0.49
Large	226.72	1.39	0.06	1.24
Control/ownership of hospital				
Government or private, collapsed category (reference)				
Government, nonfederal, public,	−151.78	−0.86	0.20	0.53
Private, non-profit, voluntary	112.93	0.69	−0.03	−0.09
Private, invest-own	104.25	1.05	0.27	0.87
Private, collapsed category	15.87	0.10	0.01	0.05
Teaching hospital	75.87	1.12	0.05	0.39
Hospital HHI based on patient flow	254.90	0.61	0.85*	2.15
Lagged (year t-1) facility-level patient summary measures				
Total number of hospitalizations	0.16***	12.43	0.00***	9.02
Percentage of cases were female	−409.79	−0.69	−0.23	−0.21
Mean length of stay	3.98	0.37	0.00	0.01
Percentage of surgical cases	2580.39***	5.43	3.99***	4.46
Mean (CMS-HCC) risk score	−51.14	−0.34	−0.55	−1.55
Age distribution (%)				
<18	293.78	0.49	2.70	1.83
18_44 (reference)				
45_64	−185.31	−0.24	2.07	1.49
65_74	597.26	1.22	2.28	1.81
75 or older	483.46	0.92	0.34	0.31
Admission type (%)				
Elective (reference)				
Emergency	−46.15	−0.40	0.21	1.04
Other	124.02	1.27	0.08	0.49
Percentage of routine discharge hospitalizations	−330.91	−1.27	−0.23	−0.58
Health insurance type (%)				
Private insurance (reference)				
Medicare	−106.02	−0.28	0.23	0.54
Medicaid	163.72	0.61	0.50	1.23
Others	−29.26	−0.14	0.15	0.57
Race (%)				
White (reference)				
Black	−2054.46*	−2.62	−0.21	−0.32
Hispanic	154.64	0.42	0.23	0.40
Other	−168.06*	−2.14	−0.07	−0.79
County-level variable				
Total number of residents in the county	−0.00	−0.17	−0.00	−1.24
Percentage of people in poverty	−2.75	−0.31	0.00	0.23
Percentage of people are Medicare beneficiaries	−258.94	−0.24	−1.00	−0.87
Percentage of people between age 16 to 64	−285.91	−0.70	−1.23	−2.03
Unemployment rate	−81.65	−0.07	−4.55*	−2.79
Per capita income	0.01	1.14	0.00	2.03
Number of anesthesiologists [MD/DO] per 10,000 residents	231.51	2.04	−0.02	−0.29
Year dummy variables				
1999.year (reference)	0.00	.	0.00	.
2000.year	78.70*	2.58	−0.01	−0.45
2001.year	90.38*	2.27	0.01	0.22
2002.year	70.76	1.02	0.07	1.33
2003.year	81.98	1.21	0.07	0.85
2004.year	141.51	1.88	−0.01	−0.12
2005.year	122.69	1.53	−0.08	−0.75
2006.year	−2.13	−0.03	−0.15	−1.38
2007.year	44.26	0.49	−0.19	−1.84
2008.year	59.28	0.45	−0.15	−1.14
2009.year	−4.30	−0.03	0.02	0.10
2010.year	−55.14	−0.29	0.01	0.07
2011.year	−183.95	−0.97	−0.06	−0.29
Constant	295.62	0.55	4.94**	3.37
N	2063		2063	
R-squared (within)	0.4010		0.2019	

*Notes*: [1] t-statistics in parentheses; *p < 0.05; **p < 0.01; ***p < 0.001; [2] Some hospital-year do not have cost-to-charge ratios; therefore, cost measure was not available; [3] interaction term between opt-out and rural hospital status was not statistically significant; therefore, main models do not include interaction terms

The results for the outpatient access models are shown in Table 3. In the model where the number of surgical procedures is the dependent variable, the estimated coefficient of the opt-out variable was positive but not statistically significant. When the dependent variable is defined as the log of procedures, the estimated coefficient of the opt-out was also positive but not statistically significant.

**Table 3:** Outpatient Access Linear and Log Models

	Total number of surgical procedures (w/o county variables)		Log of total number of surgical procedures (w/o county variables)	
	b	t	b	t
Opt out	1149.18	1.06	0.06	0.71
Lagged (year t-1) facility-level patient summary measures				
Percentage of female	10380.26	1.28	0.11	0.69
Mean (CMS-HCC) risk score	9003.39	2.17	0.24	1.45
Age distribution (%)				
<18	5126.30	0.48	0.04	0.06
18_44 (reference)				
45_64	8195.99	0.84	0.52	1.18
65_74	−29766.70	−0.86	−0.46	−1.31
75 or older	−13872.42	−1.10	0.76	1.26
Percentage of routine discharge hospitalizations	2073.23	0.52	−0.08	−0.67
Health insurance type (%)				
Private insurance				
Medicare	−1380.08	−0.68	−0.25	−1.79
Medicaid	−10119.71	−0.93	−0.27	−1.04
Others	−10856.39	−1.07	−0.46**	−5.48
Freestanding	−1043.54	−1.04	−0.08	−1.56
Year dummy variables				
1999.year (reference)				
2000.year	−16.36	−0.05	0.05***	11.74
2001.year	−923.90	−1.34	0.02*	3.63
2002.year	−876.51	−1.00	0.09***	9.77
2003.year	7386.10**	4.19	0.61***	21.00
2004.year	9061.60***	16.62	0.76***	36.50
2005.year	10213.33***	20.49	0.79***	41.31
2006.year	11373.59***	22.15	0.84***	43.80
2007.year	32466.04***	59.07	1.58***	78.96
2008.year	63228.57***	202.11	2.26***	73.62
2009.year	62817.03***	271.86	2.18***	207.52
2010.year	62829.63***	143.72	2.15***	162.03
2011.year	62947.40***	89.87	2.12***	91.22
2012.year	63165.88***	57.08	2.14***	74.13
2013.year	64712.58***	27.96	2.21***	17.61
Constant	−57093.83***	−15.05	5.98***	18.56
N	7856		7856	
Squared (within)	0.3581		0.4638	

*Note*: *p < 0.05; **p < 0.01; ***p < 0.001

To assess the robustness of our inpatient model findings, we estimated a number of models with different definitions of “surgical” discharges or different sets of covariates included in the model, as reported in Table 4. Neither early nor late opt-out states had a statistically significant impact on volumes. However, hospitals in late opt-out states (i.e. CA and CO) had a higher cost increase after state opt-out compared to hospitals in early opt-out states. When pediatric surgical discharges were removed from the facility-level total number of annual surgical discharges, the estimates of the opt-out variable coefficient remained positive but not statistically significant, in both the linear and log models. Similarly, when discharges for transplants and any craniotomy DRGs were removed from the total, or when only hip and knee procedure discharges were included, the estimates of the opt-out variable coefficient remained positive but not statistically significant in all models. In addition, dropping groups of covariates from the model specification did not materially alter the results, with one exception. In models that excluded all hospital characteristics, lagged patient flow variables, and county level variables, the estimated opt-out coefficients were negative, and statistically significant (*p* < 0.05) when the dependent variable was the number of surgical discharges.

**Table 4:** Sensitivity analyses on NIS sample (Coefficients of opt-out variable)

	Total number of surgical discharges	Log Total number of surgical discharges	Mean costs per surgical case	Log Mean costs per surgical case
Main model	39.78	0.0529	1815.3***	0.0840*
	(0.62)	(1.08)	(3.76)	(2.43)
Subgroup analysis				
Early opt-out^a^ vs control	103.9	0.0741	644.5	0.0183
	(1.50)	(1.50)	(1.42)	(0.50)
Late opt-out^b^ vs control	−185.4	0.0234	2461.0***	0.120*
	(−1.14)	(0.29)	(4.42)	(2.38)
opt-out variable * late opt-out^c^	−279.9	−0.0687	2202.9**	0.130*
	(−1.87)	(−1.16)	(3.09)	(2.38)
Alternative definitions of surgical case				
Removing cases age <18 out of total surgical discharges	39.91	0.0410	1833.5**	0.0784*
	(0.61)	(0.98)	(3.41)	(2.28)
Removing all transplant DRGs and any craniotomy DRGs	38.84	0.0535	1757.2***	0.0831*
	(0.61)	(1.09)	(3.75)	(2.39)
Include only hip and knee surgery procedures	24.12	0.00109	494.1	0.0292
	(1.55)	(0.03)	(0.63)	(1.27)
Using partial covariates				
Exclude hospital characteristics	33.71	0.0477	1839.3***	0.0762*
	(0.56)	(1.08)	(4.08)	(2.72)
Exclude hospital characteristics and county variables	6.887	0.0364	1903.8**	0.0637*
	(0.12)	(0.70)	(3.06)	(2.10)
Exclude hospital variables, county variables and t-1 year variables	−110.4*	−0.0561	1977.9**	0.0709***
	(−2.03)	(−1.18)	(2.91)	(4.71)

*Notes:* Costs were in 2011 dollar adjusted by hospital and related services CPI; ﻿*p < 0.05; **p < 0.01; ***p < 0.001

^a^Early opt out =1 for those hospitals in states opt out between 2001 and 2005 (i.e. IA, MN, NE, NH, NM, AK, KS, ND, OR, WA, MT, SD, WI)

^b^Late opt out =1 for those hospitals in states opt out between 2009 and 2010 (i.e. CA, CO)

^c^This is the coefficient for the interaction term between opt-out variable and late opt out variable. The model was conducted on whole sample to test whether state opt out in recent year had different impact on outcomes comparing those opt out in early year

In the alternative cost models, when all pediatric surgical discharges were removed, or all discharges for transplants and any craniotomy DRGs were removed, the coefficient of the opt-out variable was consistently positive and statistically significant. When only hip and knee procedure discharges were included, the estimated opt-out coefficient was positive but not statistically significant. Similarly, when groups of covariates were dropped from the model specification, the coefficient of the opt-out variable remained consistently positive and statistically significant. Point estimates suggest costs per discharge were about $1,760 to $1,980 higher (in the linear models), or about 6.6 to 8.8% higher (in log models), for facilities in opt-out states compared to non-opt-out states.

Several alternative specifications of the outpatient access model were estimated, as summarized in Table 5. In model specifications focusing on freestanding facilities, the estimated coefficient of the opt-out variable is negative and statistically significant, in both the linear and log models. This implies that the opt-out policy reduced the volume of procedures at freestanding outpatient facilities by about 310 procedures, or by about 23%. In the model limited to non-freestanding facilities, the estimated coefficient of the opt-out variable was positive but not statistically significant. When the analysis focused on selected procedures likely to require general anesthesia, the estimated coefficient of the opt-out variable was negative but not statistically significant. Finally, in model specifications dropping groups of covariates, the opt-out coefficient estimates remain positive but not statistically significant.

**Table 5:** Sensitivity and subgroup analyses on SASD sample (Coefficients of opt-out variable)

	Total number of surgical procedures	Log of total number of surgical procedures
Main model (sample includes freestanding facilities)	1149.2	0.0601
	(1.06)	(0.71)
Subgroups		
Non-freestanding	1333.9	0.129
	(1.08)	(1.93)
Freestanding	−310.2***	−0.257***
	(−15.71)	(−23.06)
Alternative definition of surgical cases		
Subset of selected procedures per facility usually requiring general anesthesia^2^	−22.84	−0.0916
	(−0.66)	(−0.76)
Using partial covariates		
Exclude t-1 year case mix variables	537.3	0.0496
	(0.34)	(0.48)

*Notes*: [1] Hospital characteristics and county variables were not available for freestanding facilities; [2] procedures with CPT code of 19301, 19302, 23410, 23412, 23420, 23430, 23470, 23472, 23473, 23474, 23700, 24300, 24341, 24342, 24363, 24370, 24371, 29827, 29882, 29883, 42821, 42826, 47562, 47563, 47600, 47605, 49505, 49507, 49520, 49521, 49525, 49587, 49650, 49651, 58541, 58542, 58543, 58544, 58545, 58546, 58550, 58552, 58553, 58554, 58570, 58571, 58572, 58573, 58670, 58671; *p < 0.05; **p < 0.01; ***p < 0.001

## Discussion

The primary intent of the opt-out laws was to increase access to anesthesia services by increasing the scope of practice of NAs and reducing the barriers to use of NAs. In turn, the hypothesis is that the reduction in barriers will increase access to surgical care. In our study, we do not find evidence to support this belief. In addition to the regression results presented in Tables 1, 2 and 3, we estimated a large number of variations of these base models (Tables 4 and 5).

Overall, the results consistently show no improvement in access to inpatient surgical care associated with the opt-out indicator. In other words, opt out was not associated with increase (or decrease) in access; the opt-out rules had no measurable effect on access. Interestingly, states choosing to opt out were associated with subsequent higher costs per inpatient —about $1,800 higher per surgery, or about 8.7%.

On the surface, the inpatient cost result seems counterintuitive, as opt-out provisions in theory allow lower-priced nurse anesthetists to perform the same services as physician anesthesiologists. However, as some recent research has shown, nurse anesthetists take longer to perform the same services. [11] As a result, despite the lower payment per unit for nurse anesthetists, the greater number of units provided may translate into higher anesthesia costs overall. Moreover, recent research suggests that surgery procedures with nurse anesthesia providers working without physician supervision have worse surgery outcomes in terms of complications requiring additional treatment. [6–8] Clearly, surgical procedures with these complications are likely to entail higher overall costs than procedures without complications. [9] Thus, the observed higher costs in opt-out states could be a result of the combined effects of these two issues.

The results for the outpatient access models are less consistent, with some model specifications indicating a reduction in access associated with opt-out status, while other model specifications suggesting no discernable change in access. It is possible that the limited number of states included in the analysis contributed to this inconsistency. Given the sensitivity of model findings to changes in model specification, the results do not provide support for the belief that opt-out policy improves access to outpatient surgical care, and may even reduce access to outpatient surgical care (among freestanding facilities).

There are some important limitations to this study. First, this is an observational study where states chose to opt out; opt-out was nota random event. There are potential unmeasured confounders associated with opt-out and outcomes. The analytic approach we used eliminates the impact of any unobservables across states that are time-invariant, but does not account for the potential impact of time-varying unobservables. It is possible that the association between opt-out status and higher surgical costs results from differences between opt-out and non-out-out states not accounted for in our analysis. Second, some opt-out states declared opt-out status toward the end of the timeline of available data, thereby providing a small number of years post opt-out years for the facility fixed-effects panel models. However, accounting for early vs. late opt-out status indicated later opt-out status was associated with greater increase in cost that the cost increase in early opt-out states, relative to non-opt-out states, but did not alter the finding of no significant improvement in access associated with opt-out. In addition, NIS randomly selected a 20% random sample of national hospitals during out study period. Some hospitals were not included in our sample or contribute fewer years of observation times which might reduce to power for the facility-level fixed-effects model. However, given the large sample, it is unlikely to be threat to our main conclusion. Finally, the opt-out status variable is a “black box” in our analysis – it does not measure to what extent either the number of nurse anesthetists or physician anesthesiologists, or their typical workloads, actually changed as a result of the implementation of the opt-out policy. However, our results suggest that, whatever the impact of opt-out on the actual supply of anesthesia services, the net impact of opt-out policy implementation was little or no impact on access to inpatient or outpatient surgical care, and an increase in the cost of inpatient surgical care.

## Conclusions

Our results do not support the hypothesis that opt-out laws improve access to inpatient surgical care or reduce its costs. Across a number of specifications for our inpatient discharges models, we find a consistent pattern of point estimates of increased costs with no discernable impact on access. Findings for our outpatient access models are less consistent, but overall, our results suggest opt-out policies were not associated with improvement in access to outpatient surgery.

## Appendix

**Table 6:** Opt out year-month for states included in our NIS and SASD sample

		Included in our sample	
State	Opt-out date	NIS	SASD
Alaska	Oct. 2003	Yes	No
Arizona	NA	Yes	No
Arkansas	NA	Yes	No
California	Jun. 2009	Yes	Yes
Colorado	Sept. 2010	Yes	Yes
Connecticut	NA	Yes	No
Florida	NA	Yes	Yes
Georgia	NA	Yes	No
Hawaii	NA	Yes	No
Illinois	NA	Yes	No
Indiana	NA	Yes	No
Iowa	Dec. 2001	Yes	No
Kansas	Apr. 2003	Yes	No
Kentucky	Apr. 2012	Yes	yes
Louisiana	NA	Yes	No
Maine	NA	Yes	No
Maryland	NA	Yes	Yes
Massachusetts	NA	Yes	No
Michigan	NA	Yes	No
Minnesota	Apr. 2002	Yes	No
Mississippi	NA	Yes	No
Missouri	NA	Yes	No
Montana	Jan. 2004	Yes	No
Nebraska	Feb. 2002	Yes	No
Nevada	NA	Yes	No
New Hampshire	Jun. 2002	Yes	No
New Jersey	NA	Yes	Yes
New Mexico	Nov. 2002	Yes	No
New York	NA	Yes	No
North Carolina	NA	Yes	No
North Dakota	Oct. 2003	Yes	No
Ohio	NA	Yes	No
Oklahoma	NA	Yes	No
Oregon	Dec. 2003	Yes	No
Pennsylvania	NA	Yes	No
Rhode Island	NA	Yes	No
South Carolina	NA	Yes	No
South Dakota	Mar. 2005	Yes	No
Tennessee	NA	Yes	No
Texas	NA	Yes	No
Utah	NA	Yes	No
Vermont	NA	Yes	No
Virginia	NA	Yes	No
Washington	Oct. 2003	Yes	No
West Virginia	NA	Yes	No
Wisconsin	Jun. 2005	Yes	No
Wyoming	NA	Yes	No

**Table 7:** Descriptive for the main outcomes in inpatient file (NIS)

Hospital state	Calendar year	Total number of surgical procedures			Log of total number of surgical procedures			Mean costs per surgical case			Log Mean costs per surgical case		
		Mean	Std	N	Mean	Std	N	Mean	Std	N	Mean	Std	N
AK	2010	352.50	318.91	2	5.60	1.07	2	24009.66	1392.48	2	10.09	0.06	2
	2011	389.00	405.88	2	5.57	1.34	2	28491.65	3365.32	2	10.25	0.12	2
AR	2004	1303.41	2157.82	29	5.36	2.50	29	5750.00	2145.57	22	8.59	0.36	22
	2005	1266.71	1802.66	24	5.86	2.01	24	6588.21	2567.39	20	8.71	0.44	20
	2006	1095.63	1635.97	24	5.41	2.20	24	8517.11	9471.11	15	8.80	0.60	15
	2007	609.68	1196.46	22	4.80	2.27	22	8088.49	6913.90	17	8.80	0.61	17
	2008	1643.27	2325.74	22	5.42	2.76	22	8132.00	3451.52	19	8.90	0.50	19
	2009	1400.42	2006.85	19	5.45	2.63	19	9725.37	3875.93	16	9.11	0.39	16
	2010	989.94	1946.63	16	5.03	2.21	16	12334.08	5633.30	15	9.34	0.41	15
	2011	1383.13	2230.16	16	5.94	1.71	16	11072.76	7855.47	12	9.17	0.50	12
AZ	1998	2099.46	2273.16	13	6.36	2.43	13	.	.	0	.	.	0
	1999	2491.25	2883.66	12	6.53	2.41	12	.	.	0	.	.	0
	2000	3115.64	3147.09	14	7.22	1.86	14	.	.	0	.	.	0
	2001	2817.27	2671.47	11	7.33	1.34	11	3893.65	1037.64	10	8.22	0.35	10
	2003	1829.69	2648.37	13	5.96	2.37	13	5872.07	2489.39	12	8.59	0.46	12
	2004	3077.92	4407.92	13	6.09	2.79	13	10111.39	11406.00	12	8.96	0.63	12
	2005	3351.11	4311.27	18	6.52	2.88	18	8077.58	2029.58	11	8.97	0.25	11
	2006	4043.00	4349.95	15	7.05	2.46	15	11028.56	2180.83	12	9.29	0.22	12
	2007	3421.53	4125.20	15	7.15	1.71	15	10193.32	2876.50	13	9.20	0.27	13
	2008	3892.56	4886.74	16	6.31	3.04	16	14195.29	11241.22	16	9.40	0.52	16
	2009	2790.31	2664.93	16	6.98	2.02	16	18316.77	14801.73	15	9.66	0.50	15
	2010	2791.87	2514.98	15	6.96	2.33	15	13640.12	4886.16	15	9.46	0.37	15
	2011	2929.69	3247.85	16	6.88	2.30	16	15769.02	4543.36	15	9.63	0.27	15
CA	1998	2121.70	2167.99	94	6.96	1.54	94	.	.	0	.	.	0
	1999	2330.86	2503.90	95	7.06	1.57	95	.	.	0	.	.	0
	2000	2218.62	2375.59	91	7.02	1.50	91	.	.	0	.	.	0
	2001	2470.48	2350.32	93	6.98	1.93	93	5827.07	2725.23	76	8.59	0.41	76
	2002	2777.43	2780.51	92	7.22	1.68	92	7099.43	2607.84	59	8.81	0.35	59
	2003	2636.85	2417.21	85	7.27	1.42	85	7754.73	3393.70	65	8.88	0.39	65
	2004	2548.71	2434.74	82	7.26	1.31	82	9071.56	4796.00	64	9.03	0.37	64
	2005	2988.06	3147.14	84	7.28	1.53	84	11114.95	5520.25	66	9.24	0.37	66
	2006	2668.75	2479.17	81	7.25	1.50	81	11296.21	5763.61	63	9.25	0.39	63
	2007	3016.74	2996.67	84	7.27	1.63	84	13775.26	7546.54	69	9.43	0.42	69
	2008	2852.38	2782.56	82	7.24	1.62	82	15579.79	8167.87	73	9.56	0.42	73
	2009	3008.26	2974.03	81	7.28	1.54	81	18361.10	11086.31	74	9.69	0.49	74
	2010	2749.08	2502.10	76	7.15	1.74	76	23461.53	17798.25	68	9.89	0.53	68
	2011	2917.49	2701.21	77	7.24	1.77	77	21961.51	8726.40	62	9.92	0.39	62
CO	1998	2118.22	2572.49	18	6.04	2.64	18	.	.	0	.	.	0
	1999	2000.06	2618.33	17	5.89	2.74	17	.	.	0	.	.	0
	2000	1793.05	2551.32	21	5.54	2.72	21	.	.	0	.	.	0
	2001	1959.31	2362.98	16	6.19	2.47	16	5497.33	1593.74	11	8.57	0.31	11
	2002	2199.06	3192.30	18	5.45	3.06	18	6031.03	973.12	11	8.69	0.15	11
	2003	1775.06	2425.82	18	5.80	2.57	18	6982.06	1906.24	15	8.82	0.27	15
	2004	2233.00	2843.94	18	6.11	2.73	18	7802.34	1956.12	15	8.93	0.26	15
	2005	2131.39	2924.85	18	5.55	3.19	18	8516.21	3115.39	16	8.96	0.47	16
	2006	1793.11	2699.90	18	5.41	3.01	18	10108.59	4811.35	16	9.06	0.71	16
	2007	2440.00	3039.16	18	6.19	2.65	18	12647.78	5101.15	17	9.38	0.35	17
	2008	2688.25	3054.51	16	6.38	2.66	16	17127.62	11548.88	15	9.60	0.53	15
	2009	2947.40	2995.45	15	7.03	1.78	15	17669.03	5501.60	15	9.74	0.30	15
	2010	2369.47	2769.70	15	6.05	2.82	15	18367.56	6221.43	15	9.74	0.45	15
	2011	2088.00	2184.81	18	6.52	2.17	18	19800.23	5267.59	17	9.86	0.26	17
CT	1998	1629.14	1010.77	7	7.25	0.57	7	.	.	0	.	.	0
	1999	3696.33	4394.93	6	7.74	1.03	6	.	.	0	.	.	0
	2000	3374.83	3147.44	6	7.73	1.00	6	.	.	0	.	.	0
	2001	4053.43	4358.35	7	7.81	1.11	7	5865.99	994.65	7	8.66	0.17	7
	2002	4643.25	4299.35	8	8.09	0.90	8	6811.11	1064.44	8	8.81	0.17	8
	2003	3546.83	3122.36	6	7.77	1.08	6	7541.01	415.77	5	8.93	0.06	5
	2004	3368.30	2431.76	10	7.91	0.68	10	7924.88	1407.79	10	8.96	0.19	10
	2005	3519.13	4567.21	8	7.57	1.15	8	9390.75	1255.57	7	9.14	0.13	7
	2006	4116.50	4043.90	10	7.95	0.92	10	10223.72	1779.58	10	9.22	0.18	10
	2007	3505.22	2348.92	9	7.96	0.69	9	11712.55	2130.87	9	9.35	0.19	9
	2008	5080.00	5647.94	6	7.96	1.21	6	14300.30	2949.97	6	9.55	0.22	6
	2009	4138.86	3933.42	7	7.92	1.01	7	14035.06	3406.36	7	9.53	0.21	7
	2010	4098.14	3510.21	7	8.01	0.88	7	15497.02	3034.58	7	9.63	0.21	7
	2011	3284.75	3282.91	8	7.72	0.95	8	15951.15	2377.51	7	9.67	0.16	7
FL	1998	2554.55	2636.13	106	7.08	1.79	106	.	.	0	.	.	0
	1999	2614.08	3237.07	97	6.95	1.91	97	.	.	0	.	.	0
	2000	2775.69	3191.06	55	6.99	2.01	55	.	.	0	.	.	0
	2001	3008.64	3417.63	55	7.13	1.88	55	5252.91	1226.49	42	8.54	0.23	42
	2002	4004.06	3855.04	51	7.54	1.79	51	6029.14	1587.95	44	8.67	0.28	44
	2003	3290.84	3462.90	58	7.11	2.10	58	7150.79	2201.82	48	8.83	0.31	48
	2004	3335.84	4102.03	55	7.29	1.79	55	8097.76	2484.26	48	8.96	0.30	48
	2005	3245.45	4702.04	51	7.38	1.49	51	9212.34	4003.89	43	9.07	0.31	43
	2006	3552.69	5111.64	51	7.09	1.99	51	10266.39	5588.96	40	9.13	0.44	40
	2007	4014.08	4663.80	50	7.49	1.79	50	10307.93	3107.63	45	9.19	0.38	45
	2008	4171.35	5590.79	49	7.37	1.85	49	11937.08	4949.32	45	9.33	0.32	45
	2009	3520.84	5131.49	50	6.99	2.19	50	12483.18	3539.47	44	9.39	0.29	44
	2010	3629.66	4004.72	44	7.45	1.62	44	16103.68	6455.51	41	9.63	0.33	41
	2011	3433.17	4102.18	46	7.36	1.67	46	16100.65	8814.13	41	9.62	0.32	41
GA	1998	1105.33	1664.79	111	5.61	2.07	111	.	.	0	.	.	0
	1999	1212.11	2133.15	97	5.50	2.09	97	.	.	0	.	.	0
	2000	1449.82	2784.19	57	5.37	2.36	57	.	.	0	.	.	0
	2001	1428.32	2214.31	56	5.67	2.28	56	4565.10	1239.12	34	8.39	0.26	34
	2002	1709.04	3150.41	56	5.41	2.50	56	5581.48	1793.79	33	8.48	0.86	33
	2003	1674.18	3428.28	50	5.55	2.27	50	5784.26	1921.51	24	8.60	0.38	24
	2004	1540.32	2951.55	50	5.50	2.35	50	7671.76	4026.51	30	8.75	0.92	30
	2005	1675.70	2470.76	46	5.86	2.40	46	8251.12	2680.96	34	8.97	0.30	34
	2006	1899.24	3001.79	42	5.96	2.29	42	8825.86	2360.98	34	9.05	0.29	34
	2007	2263.03	3518.61	38	6.04	2.43	38	9529.58	3019.18	27	9.12	0.31	27
	2008	1787.58	2382.77	33	5.78	2.48	33	11587.75	4943.59	24	9.28	0.41	24
	2009	1902.58	3117.79	38	5.61	2.51	38	13192.84	7595.41	32	9.39	0.42	32
	2010	1555.05	2097.80	39	5.82	2.29	39	15261.72	11840.58	36	9.48	0.54	36
	2011	1531.57	2266.45	35	5.60	2.50	35	14821.94	5090.16	33	9.55	0.33	33
HI	1998	1140.75	1091.51	4	6.30	1.77	4	.	.	0	.	.	0
	1999	1589.67	1095.95	3	7.12	0.96	3	.	.	0	.	.	0
	2000	1775.67	1192.74	3	7.21	1.04	3	.	.	0	.	.	0
	2001	2012.00	1189.55	3	7.42	0.83	3	.	.	0	.	.	0
	2002	1580.80	1081.55	5	6.03	3.39	5	.	.	0	.	.	0
	2003	915.40	553.97	5	6.64	0.69	5	8995.32	3738.26	4	9.04	0.43	4
	2004	1206.40	1135.34	5	6.63	1.19	5	8027.69	3433.08	5	8.92	0.41	5
	2005	1307.75	1067.76	4	6.91	0.86	4	7571.95	1720.19	3	8.91	0.25	3
	2006	1101.75	779.11	4	6.74	0.90	4	10315.77	2827.16	4	9.21	0.27	4
	2007	1771.25	1367.42	4	7.15	1.08	4	10007.89	658.75	3	9.21	0.07	3
	2008	1147.33	442.97	3	6.99	0.44	3	17131.34	9267.88	3	9.66	0.50	3
	2009	2796.00	.	1	7.94	.	1	19871.55	.	1	9.90	.	1
	2010	1926.75	1060.69	4	7.39	0.75	4	15053.38	7084.85	4	9.55	0.42	4
	2011	.	.	0	.	.	0	.	.	0	.	.	0
IA	1998	849.11	1794.65	53	5.25	1.68	53	.	.	0	.	.	0
	1999	1059.67	2052.33	54	5.30	2.01	54	.	.	0	.	.	0
	2000	1163.16	2206.46	51	5.42	1.92	51	.	.	0	.	.	0
	2001	927.62	1832.94	37	5.32	1.76	37	4748.16	1232.84	25	8.44	0.22	25
	2002	1080.00	2054.53	28	5.36	1.99	28	5224.04	1593.30	16	8.53	0.25	16
	2003	1079.26	2278.38	27	5.20	2.03	27	5596.97	1007.46	17	8.61	0.18	17
	2004	984.92	2188.72	26	4.85	2.24	26	6665.14	2304.46	14	8.76	0.32	14
	2005	912.25	2196.46	28	4.92	2.21	28	7447.16	2289.44	19	8.88	0.28	19
	2006	933.48	1982.12	29	5.04	2.16	29	7978.59	1480.96	21	8.97	0.19	21
	2007	1014.59	2045.78	27	5.04	2.24	27	8824.55	1496.89	19	9.07	0.19	19
	2008	572.59	1409.53	27	4.46	2.11	27	11368.58	3182.80	24	9.30	0.27	24
	2009	634.40	1439.88	25	4.54	2.10	25	16469.25	11479.62	24	9.56	0.50	24
	2010	784.69	1891.69	26	4.50	2.26	26	16279.03	7491.64	25	9.63	0.36	25
	2011	622.75	1059.96	24	4.63	2.15	24	17281.74	5435.92	24	9.71	0.29	24
IL	1998	1915.22	2039.10	74	6.80	1.50	74	.	.	0	.	.	0
	1999	2039.42	2380.00	69	6.72	1.67	69	.	.	0	.	.	0
	2000	2164.01	2692.61	68	6.70	1.68	68	.	.	0	.	.	0
	2001	1943.46	2069.21	65	6.85	1.48	65	5717.47	2415.54	57	8.60	0.31	57
	2002	1987.61	2023.36	46	6.67	1.83	46	6270.24	2823.11	40	8.67	0.37	40
	2003	2138.19	2206.29	42	6.60	2.07	42	6931.04	1482.53	36	8.82	0.23	36
	2004	2040.20	2539.70	40	6.44	2.20	40	8211.13	2369.53	33	8.98	0.26	33
	2005	1917.23	2353.27	43	6.46	1.79	43	8605.82	2595.79	39	9.02	0.28	39
	2006	1980.63	2282.61	40	6.51	1.90	40	10421.62	4419.12	38	9.18	0.36	38
	2007	2510.68	3465.31	41	6.69	1.91	41	12169.86	3928.83	39	9.36	0.29	39
	2008	2012.68	3045.48	44	6.18	2.23	44	15546.74	14632.14	40	9.51	0.43	40
	2009	2156.28	3176.05	40	6.23	2.45	40	14185.09	5070.47	38	9.51	0.30	38
	2010	1666.00	2066.70	44	6.21	2.04	44	19032.69	14877.23	44	9.73	0.42	44
	2011	2171.50	2927.36	40	6.22	2.42	40	18344.68	5021.59	40	9.78	0.27	40
IN	2003	1649.25	2370.52	24	6.24	1.65	24	6884.33	2170.24	19	8.79	0.31	19
	2004	1591.21	2183.46	24	6.45	1.43	24	7734.78	1865.94	19	8.92	0.25	19
	2005	1972.68	3039.02	25	6.62	1.45	25	9288.90	5964.20	22	9.02	0.44	22
	2006	1737.81	2383.75	26	6.46	1.55	26	10825.71	8876.06	25	9.15	0.46	25
	2007	1691.65	1601.06	26	6.68	1.50	26	11002.11	3678.46	23	9.24	0.41	23
	2008	2315.56	3771.24	27	6.66	1.62	27	11053.83	4715.52	24	9.19	0.57	24
	2009	1859.04	2602.55	27	6.54	1.58	27	13065.81	5177.26	25	9.36	0.57	25
	2010	2363.33	3663.95	27	6.75	1.71	27	16362.84	8542.15	27	9.58	0.56	27
	2011	1926.80	3586.77	30	6.30	1.80	30	15668.48	5960.95	30	9.57	0.47	30
KS	1998	812.74	2029.40	50	4.62	2.42	50	.	.	0	.	.	0
	1999	761.69	1638.91	51	4.86	2.16	51	.	.	0	.	.	0
	2000	1055.36	2132.07	47	5.16	2.18	47	.	.	0	.	.	0
	2001	1018.44	2134.58	32	4.86	2.43	32	4036.40	1532.23	21	8.24	0.35	21
	2002	851.18	2038.20	28	4.83	2.04	28	4618.32	1501.20	17	8.38	0.37	17
	2003	1161.13	2631.74	24	4.64	2.72	24	5552.28	1640.88	18	8.56	0.39	18
	2004	989.04	2316.78	23	4.40	2.70	23	6669.50	1367.83	11	8.79	0.20	11
	2005	592.18	991.01	17	4.52	2.53	17	6394.52	1722.91	9	8.73	0.26	9
	2006	633.87	1421.98	23	3.95	2.68	23	7227.04	1887.91	15	8.85	0.27	15
	2007	724.00	1212.85	21	4.63	2.56	21	7258.34	2144.50	15	8.84	0.32	15
	2008	617.63	1486.77	24	4.08	2.31	24	10000.85	2734.45	21	9.17	0.29	21
	2009	761.48	1994.03	23	4.12	2.41	23	12874.11	4391.28	22	9.41	0.32	22
	2010	757.86	1782.16	22	4.65	2.26	22	11578.77	3946.00	22	9.30	0.35	22
	2011	674.60	1820.44	25	4.20	2.51	25	14580.75	6887.72	23	9.48	0.49	23
KY	2000	1372.93	2559.16	30	5.44	2.45	30	.	.	0	.	.	0
	2001	1297.93	1838.57	28	5.42	2.62	28	4036.06	1608.75	24	8.23	0.38	24
	2002	1518.56	2360.15	32	5.81	2.20	32	4568.06	1353.64	26	8.39	0.28	26
	2003	1365.83	2290.43	29	5.61	2.29	29	5392.58	1978.43	24	8.52	0.39	24
	2004	1184.62	1804.00	26	5.52	2.20	26	6320.87	2287.17	21	8.70	0.32	21
	2005	1503.11	2660.09	27	5.52	2.30	27	6372.96	2318.52	21	8.70	0.35	21
	2006	1841.32	2983.14	25	5.83	2.31	25	7091.80	3002.84	19	8.74	0.61	19
	2007	1333.26	2458.16	27	5.45	2.41	27	7402.65	2385.76	20	8.70	1.10	20
	2008	1256.75	2090.24	24	5.12	2.78	24	10213.45	3879.04	21	9.16	0.41	21
	2009	1966.50	2974.51	20	5.97	2.47	20	13063.02	10692.82	18	9.33	0.48	18
	2010	1798.45	2655.97	22	5.68	2.66	22	10951.32	5302.18	17	9.14	0.66	17
	2011	1669.30	2789.48	20	5.18	2.85	20	12887.79	3244.17	19	9.43	0.25	19
LA	2008	1181.38	1549.66	26	5.77	2.17	26	9885.81	3275.27	21	9.13	0.43	21
	2009	1156.17	1381.91	24	5.53	2.44	24	11361.36	5101.90	19	9.23	0.50	19
	2010	1306.40	2135.42	25	5.50	2.38	25	12227.67	9150.26	22	9.20	0.66	22
	2011	1416.08	2051.03	25	5.76	2.50	25	13909.64	3844.48	19	9.50	0.28	19
MA	1998	3040.06	3560.46	17	7.45	1.16	17	.	.	0	.	.	0
	1999	2668.40	2537.06	15	7.38	1.20	15	.	.	0	.	.	0
	2000	3492.94	3555.67	16	7.52	1.40	16	.	.	0	.	.	0
	2001	3410.13	3651.13	16	7.42	1.46	16	8413.99	8440.90	12	8.81	0.60	12
	2002	3442.00	3733.66	16	7.58	1.23	16	5895.69	1241.55	9	8.66	0.21	9
	2003	2534.50	2758.85	14	7.08	1.61	14	9383.10	9013.23	11	8.94	0.57	11
	2004	3447.76	4828.80	21	7.28	1.56	21	8081.33	2197.82	18	8.96	0.26	18
	2005	2954.87	3051.40	23	7.25	1.52	23	8492.44	2342.80	21	9.02	0.24	21
	2006	1984.33	2277.51	21	6.67	1.72	21	13118.39	10793.59	18	9.32	0.49	18
	2007	2370.05	3722.28	22	6.76	1.57	22	14091.16	11434.36	19	9.41	0.45	19
	2008	3722.93	5545.08	15	7.41	1.38	15	13294.67	3883.90	15	9.46	0.27	15
	2009	2367.93	2574.38	14	7.19	1.18	14	13486.80	3630.77	13	9.48	0.22	13
	2010	2990.86	3120.01	14	7.20	1.64	14	15322.99	4759.14	13	9.60	0.27	13
	2011	3654.36	5632.16	11	7.50	1.25	11	16360.45	4950.32	11	9.67	0.27	11
MD	1998	2973.22	2512.32	32	7.48	1.26	32	.	.	0	.	.	0
	1999	3517.17	3578.67	23	7.74	0.98	23	.	.	0	.	.	0
	2000	3436.38	2713.03	13	7.82	0.87	13	.	.	0	.	.	0
	2001	4083.83	3087.75	12	7.99	0.90	12	5106.37	949.44	11	8.52	0.20	11
	2002	3958.43	2949.12	14	7.97	0.89	14	5944.15	1251.39	14	8.67	0.21	14
	2003	4126.85	2696.23	13	8.03	0.92	13	6257.75	1462.82	12	8.72	0.23	12
	2004	3628.25	2564.61	12	7.92	0.82	12	7709.09	1974.82	12	8.92	0.26	12
	2005	4483.27	2663.02	11	8.19	0.78	11	6998.57	929.72	9	8.85	0.13	9
	2006	3669.08	3385.82	12	7.83	0.93	12	10206.58	2110.85	12	9.21	0.21	12
	2007	3757.83	2852.34	12	7.71	1.47	12	10210.26	2821.95	11	9.20	0.25	11
	2008	4213.83	3314.87	12	7.72	1.58	12	12733.43	3290.62	11	9.42	0.25	11
	2009	5319.13	3533.63	8	8.25	0.99	8	11155.02	2571.94	7	9.30	0.23	7
	2010	3738.00	3071.34	9	7.83	1.02	9	13287.21	3706.27	9	9.47	0.25	9
	2011	5494.45	5052.51	11	8.11	1.18	11	15155.95	6048.65	11	9.57	0.34	11
ME	1999	1784.18	3545.32	11	6.29	1.69	11	.	.	0	.	.	0
	2000	798.90	762.74	10	6.37	0.80	10	.	.	0	.	.	0
	2001	866.22	841.53	9	6.27	1.15	9	5899.08	784.50	7	8.67	0.14	7
	2002	672.43	981.72	7	5.90	1.09	7	5421.55	1387.84	5	8.57	0.26	5
	2007	455.22	486.18	9	5.54	1.38	9	.	.	0	.	.	0
	2008	577.86	579.34	7	5.60	1.66	7	.	.	0	.	.	0
	2009	390.33	415.61	6	5.30	1.50	6	.	.	0	.	.	0
	2010	315.29	222.46	7	5.55	0.70	7	.	.	0	.	.	0
	2011	357.14	324.29	7	5.53	0.93	7	.	.	0	.	.	0
MI	2001	2437.66	2918.32	29	6.76	1.93	29	4394.89	1119.15	25	8.36	0.26	25
	2002	2678.46	4960.38	28	6.60	1.81	28	5052.02	930.37	23	8.51	0.19	23
	2003	2652.76	5418.02	21	6.49	1.98	21	5558.11	1567.72	19	8.58	0.34	19
	2004	1868.60	2617.99	20	6.67	1.49	20	6615.06	1467.27	14	8.77	0.22	14
	2005	2054.60	3654.68	25	6.32	1.88	25	7694.10	2427.38	18	8.91	0.25	18
	2006	1846.32	2825.92	22	6.31	1.76	22	8437.68	1965.92	16	9.01	0.24	16
	2007	1972.32	3272.58	25	6.07	2.19	25	14935.53	21699.29	18	9.29	0.62	18
	2008	2012.11	2955.49	27	6.06	2.15	27	12527.02	3229.72	22	9.40	0.26	22
	2009	1877.15	2753.51	27	6.14	2.01	27	13048.33	4404.20	23	9.43	0.32	23
	2010	1791.81	2966.56	26	6.00	2.00	26	15506.22	6379.94	21	9.58	0.37	21
	2011	2327.68	4861.48	25	6.03	2.04	25	18401.67	7173.68	21	9.75	0.37	21
MN	2001	1593.49	2871.27	37	5.55	2.41	37	4813.53	1573.53	32	8.44	0.29	32
	2002	1396.35	2597.47	31	5.69	2.13	31	5584.56	2184.26	24	8.56	0.35	24
	2003	1732.46	3046.11	26	5.47	2.43	26	5958.37	2351.46	21	8.60	0.52	21
	2004	1423.04	2633.96	27	5.41	2.27	27	7116.96	1616.54	16	8.85	0.23	16
	2005	1724.23	4070.53	26	5.26	2.47	26	8187.22	3287.80	13	8.96	0.31	13
	2006	1765.89	3706.60	27	5.71	2.20	27	9131.42	2342.33	14	9.09	0.24	14
	2007	1449.47	2417.68	30	5.76	2.12	30	10200.21	2776.88	21	9.20	0.27	21
	2008	1577.71	2661.17	28	5.76	2.30	28	13660.25	6083.18	27	9.45	0.38	27
	2009	1291.23	3283.06	30	5.10	2.33	30	13710.38	4154.01	29	9.49	0.27	29
	2010	1625.16	3344.31	25	5.64	2.31	25	15024.33	4565.17	25	9.58	0.27	25
	2011	1541.65	2585.63	26	5.52	2.46	26	19900.57	8287.35	26	9.83	0.37	26
MO	1998	1384.87	1789.82	38	6.36	1.44	38	.	.	0	.	.	0
	1999	2346.20	3602.97	35	6.36	2.20	35	.	.	0	.	.	0
	2000	2107.66	2901.98	38	6.15	2.43	38	.	.	0	.	.	0
	2001	1291.10	1666.76	21	6.05	1.78	21	4983.54	1252.15	17	8.48	0.27	17
	2002	2832.67	4342.81	18	6.73	1.95	18	6225.39	1784.52	15	8.70	0.29	15
	2003	2191.96	4034.85	25	5.93	2.52	25	7080.02	2561.44	17	8.80	0.36	17
	2004	2332.17	4237.76	24	6.06	2.40	24	7334.60	2871.31	18	8.78	0.64	18
	2005	2535.93	3440.55	29	6.22	2.53	29	9209.76	3689.95	23	9.06	0.37	23
	2006	2255.48	2573.19	27	6.63	1.98	27	10243.57	3248.81	23	9.19	0.31	23
	2007	1200.50	1719.23	28	5.43	2.50	28	9125.08	2427.47	22	9.09	0.25	22
	2008	1814.26	2165.41	27	6.08	2.39	27	11214.30	3697.32	26	9.27	0.35	26
	2009	1825.19	2926.66	27	5.98	2.48	27	11866.57	4808.88	27	9.31	0.38	27
	2010	2080.37	2909.41	27	5.99	2.55	27	15732.51	5576.50	25	9.60	0.35	25
	2011	2894.96	4411.42	24	6.59	2.16	24	14798.95	3745.77	24	9.57	0.25	24
MS	2010	1298.35	2104.14	17	5.23	2.66	17	10981.42	4400.66	14	9.19	0.58	14
	2011	1380.00	2200.31	19	4.68	3.22	19	14807.05	15310.98	15	9.32	0.72	15
MT	2009	996.00	1867.11	7	4.84	2.81	7	13118.94	3808.17	7	9.44	0.30	7
	2010	910.38	1783.19	8	5.23	2.17	8	16332.89	4141.19	8	9.68	0.23	8
	2011	452.80	821.81	5	4.29	2.75	5	14200.83	3843.96	5	9.53	0.27	5
NC	2000	2442.09	3148.80	35	6.90	1.56	35	.	.	0	.	.	0
	2001	2527.59	3508.26	34	6.61	2.03	34	4601.95	1324.24	29	8.40	0.26	29
	2002	1780.33	2997.44	33	6.26	2.05	33	5084.64	1048.73	21	8.52	0.19	21
	2003	2282.53	3427.70	38	6.56	2.05	38	6019.75	2049.56	29	8.66	0.29	29
	2004	2433.35	3271.46	34	6.69	1.94	34	6036.25	1159.98	23	8.69	0.21	23
	2005	2701.42	3844.66	31	6.61	2.23	31	8377.00	4083.28	26	8.96	0.34	26
	2006	2969.15	4299.20	27	6.45	2.56	27	8933.45	2630.25	22	9.06	0.26	22
	2007	2806.31	4039.36	29	6.49	2.44	29	11426.09	5569.56	24	9.27	0.36	24
	2008	2356.43	3188.32	28	6.72	1.86	28	11853.39	4375.17	25	9.33	0.30	25
	2009	2685.00	4077.54	29	6.47	2.18	29	13271.58	6430.74	27	9.42	0.34	27
	2010	2947.85	4413.61	26	6.71	2.10	26	14580.08	7988.63	25	9.49	0.44	25
	2011	2290.96	4080.40	27	5.98	2.43	27	14709.72	3748.94	23	9.57	0.24	23
ND	2011	766.00	1381.47	4	5.01	2.15	4	12425.76	5162.41	4	9.36	0.44	4
NE	2001	562.05	1222.06	20	3.93	2.37	20	.	.	0	.	.	0
	2002	533.53	1360.54	19	4.23	2.06	19	.	.	0	.	.	0
	2003	570.63	1396.58	16	4.25	2.12	16	.	.	0	.	.	0
	2004	713.53	1827.72	19	3.51	2.64	19	.	.	0	.	.	0
	2005	510.33	1040.81	15	4.45	2.08	15	.	.	0	.	.	0
	2006	801.33	1110.69	15	4.72	2.56	15	9960.69	3433.49	9	9.15	0.35	9
	2007	639.33	1698.76	15	3.78	2.36	15	9642.63	2243.55	7	9.15	0.26	7
	2008	813.94	1701.11	18	4.48	2.21	18	10768.75	3504.14	15	9.24	0.28	15
	2009	911.50	1960.16	16	5.03	2.01	16	12443.33	2605.78	16	9.41	0.22	16
	2010	719.79	1024.04	14	5.11	2.07	14	15368.49	4066.29	12	9.61	0.26	12
	2011	440.38	700.19	13	4.26	2.29	13	17086.93	6536.34	12	9.67	0.41	12
NH	2003	789.67	954.14	6	6.01	1.25	6	8073.64	1506.33	3	8.98	0.19	3
	2004	1587.75	2546.31	8	6.59	1.24	8	9241.90	1720.66	8	9.11	0.20	8
	2005	1873.50	2401.78	10	6.83	1.29	10	11076.00	1891.01	9	9.30	0.17	9
	2006	1634.78	2675.53	9	6.48	1.39	9	13310.25	1558.80	6	9.49	0.12	6
	2007	2340.38	2876.16	8	6.99	1.45	8	13343.93	2310.80	6	9.49	0.18	6
	2008	1736.75	1766.40	4	6.90	1.32	4	18555.85	2540.10	4	9.82	0.13	4
	2009	2649.80	3523.12	5	7.14	1.43	5	18094.97	4044.47	4	9.78	0.26	4
NJ	1998	4104.00	3571.86	17	7.94	0.93	17	.	.	0	.	.	0
	1999	2952.24	2252.43	17	7.72	0.76	17	.	.	0	.	.	0
	2000	3773.38	3415.28	16	7.94	0.78	16	.	.	0	.	.	0
	2001	3084.86	2365.69	14	7.80	0.70	14	5106.36	1349.19	7	8.51	0.26	7
	2002	4277.36	4395.13	14	7.95	0.92	14	5692.50	1737.38	10	8.61	0.27	10
	2003	3127.31	1839.55	16	7.86	0.68	16	6580.77	3003.24	8	8.73	0.34	8
	2004	4299.23	3217.16	22	8.11	0.76	22	8103.54	4357.98	14	8.91	0.41	14
	2005	3516.14	3766.92	22	7.66	1.26	22	7529.17	1095.34	13	8.92	0.14	13
	2006	3169.64	2372.15	22	7.55	1.53	22	14652.67	12027.58	10	9.40	0.60	10
	2007	3520.62	3285.25	21	7.43	1.78	21	12707.51	8816.48	17	9.33	0.43	17
	2008	3853.06	3194.61	16	7.26	2.26	16	15692.37	12536.29	14	9.48	0.55	14
	2009	3955.79	3702.55	14	7.80	1.26	14	15506.79	9760.97	14	9.54	0.45	14
	2010	4224.00	2393.15	14	8.19	0.59	14	17528.16	12964.92	14	9.63	0.49	14
	2011	3132.64	2052.52	14	7.58	1.44	14	22222.96	18062.96	13	9.81	0.58	13
NM	2009	655.71	527.19	7	5.84	1.68	7	10775.84	4235.77	7	9.24	0.31	7
	2010	696.56	905.38	9	5.05	2.62	9	10790.77	3921.69	7	9.23	0.35	7
	2011	1492.38	2788.29	8	6.12	1.75	8	14892.99	7600.51	6	9.48	0.58	6
NV	2002	2201.25	3100.49	8	6.09	2.68	8	6966.88	1059.68	5	8.84	0.17	5
	2003	2253.29	2768.57	7	5.18	3.54	7	6787.26	2361.39	5	8.76	0.41	5
	2004	4368.00	3669.72	5	7.89	1.26	5	8673.05	2100.31	5	9.05	0.23	5
	2005	1277.33	2140.73	6	5.72	2.14	6	8660.39	3033.09	4	9.03	0.32	4
	2006	2686.33	3136.24	9	6.35	2.91	9	8005.18	3232.06	6	8.89	0.54	6
	2007	2927.75	3811.88	8	6.41	2.97	8	12916.99	3347.11	8	9.44	0.26	8
	2008	1591.73	2078.72	11	5.77	2.85	11	11950.01	3285.93	9	9.36	0.27	9
	2009	2274.13	3016.42	8	6.82	1.60	8	13108.50	2823.15	8	9.45	0.27	8
	2010	2738.36	3289.76	11	6.60	2.20	11	15425.89	4224.43	10	9.61	0.25	10
	2011	2290.71	2654.78	7	6.59	2.05	7	24908.23	20308.00	7	9.94	0.58	7
NY	1998	2132.19	2436.21	52	6.66	2.18	52	.	.	0	.	.	0
	1999	2416.58	2747.45	45	7.07	1.61	45	.	.	0	.	.	0
	2000	2853.49	3129.06	45	7.42	1.11	45	.	.	0	.	.	0
	2001	3242.28	4043.79	43	7.33	1.44	43	5590.42	2621.72	37	8.54	0.42	37
	2002	2766.66	3293.78	44	7.18	1.48	44	6597.20	3619.77	36	8.68	0.47	36
	2003	3427.55	3942.44	42	7.16	2.07	42	6155.87	2491.87	37	8.65	0.38	37
	2004	2883.38	3912.49	63	7.06	1.84	63	7947.98	4762.75	52	8.86	0.46	52
	2005	3028.80	3697.53	64	7.10	1.94	64	7771.71	3128.96	53	8.88	0.42	53
	2006	3020.45	4156.70	62	7.14	1.69	62	9034.17	3718.11	55	9.02	0.44	55
	2007	3196.18	3713.86	55	7.25	1.76	55	10117.92	4292.29	49	9.15	0.39	49
	2008	3437.11	4719.26	38	6.87	2.47	38	15607.42	20432.51	37	9.37	0.68	37
	2009	3323.08	4009.90	38	7.52	1.11	38	13472.88	4279.28	32	9.46	0.32	32
	2010	3114.32	3725.60	38	7.22	1.79	38	13776.02	6008.00	33	9.46	0.35	33
	2011	3588.00	6240.09	40	7.04	2.02	40	15140.17	5564.67	36	9.56	0.36	36
OH	2002	2527.00	3307.23	37	7.06	1.32	37	5908.96	1303.04	28	8.66	0.22	28
	2003	2587.85	2724.95	33	7.00	1.95	33	7036.82	1924.44	29	8.81	0.35	29
	2004	2527.94	2588.13	34	7.28	1.19	34	7707.24	1442.44	32	8.93	0.18	32
	2005	2737.21	3092.45	34	7.17	1.42	34	8634.29	1551.10	26	9.05	0.19	26
	2006	2699.14	3322.00	35	6.95	1.62	35	10032.29	4909.26	31	9.16	0.30	31
	2007	2786.47	2714.96	36	7.32	1.30	36	10916.53	2376.28	30	9.28	0.21	30
	2008	2935.29	3930.51	34	7.02	1.65	34	11690.49	2291.54	31	9.35	0.20	31
	2009	2953.63	4831.75	40	7.01	1.57	40	13534.72	4883.91	40	9.46	0.33	40
	2010	2437.24	2835.55	38	6.80	1.79	38	16032.38	8480.62	38	9.61	0.36	38
	2011	2176.49	2657.24	35	6.85	1.48	35	16131.09	4427.39	35	9.65	0.26	35
OK	2005	973.03	2084.27	38	4.45	2.69	38	8844.59	9644.56	29	8.83	0.61	29
	2006	1382.65	2751.57	37	5.02	2.57	37	7603.12	6893.63	28	8.73	0.58	28
	2007	1165.94	2019.20	33	4.99	2.68	33	9764.43	8765.00	27	9.03	0.47	27
	2008	907.56	2022.08	34	4.79	2.56	34	9381.34	5931.77	29	8.99	0.56	29
	2009	1104.82	2458.02	33	5.00	2.39	33	12639.34	11496.96	24	9.24	0.58	24
	2010	1314.17	2576.75	29	5.30	2.37	29	14013.95	13477.54	27	9.30	0.65	27
	2011	823.24	1745.55	29	4.93	2.36	29	14412.94	9515.59	25	9.40	0.59	25
OR	1998	1143.94	1866.50	18	5.91	1.85	18	.	.	0	.	.	0
	1999	1732.29	2261.67	17	6.24	2.11	17	.	.	0	.	.	0
	2000	1630.78	2488.82	18	6.20	2.00	18	.	.	0	.	.	0
	2001	1424.00	2134.01	19	6.03	1.91	19	.	.	0	.	.	0
	2002	2397.56	4019.29	16	6.43	1.77	16	.	.	0	.	.	0
	2003	1203.31	1619.97	16	5.98	1.88	16	7091.79	2519.14	12	8.82	0.32	12
	2004	2312.77	3800.60	13	6.80	1.39	13	.	.	0	.	.	0
	2005	1246.38	2012.70	16	6.18	1.52	16	.	.	0	.	.	0
	2006	2257.20	3245.54	15	6.41	1.89	15	11521.55	2797.04	13	9.32	0.24	13
	2007	1826.40	2653.57	15	6.58	1.46	15	11130.62	2044.44	14	9.30	0.19	14
	2008	1935.00	3184.08	17	6.25	1.83	17	14682.31	3517.72	16	9.57	0.23	16
	2009	2430.36	3640.99	14	6.61	1.81	14	15175.93	3525.53	14	9.60	0.23	14
	2010	1850.64	3201.19	14	6.08	2.35	14	17091.32	4036.51	13	9.72	0.23	13
	2011	1630.07	2867.50	15	5.78	2.36	15	19629.93	8637.12	14	9.80	0.43	14
PA	1998	2004.91	1946.07	47	6.96	1.39	47	.	.	0	.	.	0
	1999	2282.86	2820.36	42	7.14	1.15	42	.	.	0	.	.	0
	2000	2262.83	2459.99	42	7.12	1.22	42	.	.	0	.	.	0
	2001	2591.44	3177.39	41	7.15	1.38	41	.	.	0	.	.	0
	2002	2357.03	2555.95	40	7.21	1.16	40	.	.	0	.	.	0
	2003	2845.40	4667.72	40	7.13	1.49	40	.	.	0	.	.	0
	2008	2856.68	4943.77	38	6.67	2.04	38	.	.	0	.	.	0
	2009	2776.63	3268.88	35	6.71	2.06	35	.	.	0	.	.	0
	2010	2782.76	3753.00	41	6.98	1.61	41	.	.	0	.	.	0
	2011	2701.51	4629.25	41	6.84	1.67	41	.	.	0	.	.	0
RI	2001	1331.00	.	1	7.19	.	1	4444.86	.	1	8.40	.	1
	2002	1815.00	.	1	7.50	.	1	7208.26	.	1	8.88	.	1
	2003	4083.75	4135.39	4	7.97	0.91	4	10011.33	.	1	9.21	.	1
	2004	3034.67	1967.73	3	7.88	0.64	3	5181.99	777.22	3	8.55	0.15	3
	2005	2790.67	2302.59	3	7.72	0.77	3	6608.93	2198.37	2	8.77	0.34	2
	2006	1776.33	484.87	3	7.45	0.30	3	8928.95	1255.04	3	9.09	0.15	3
	2007	3505.00	3163.60	2	7.90	1.07	2	8270.36	3117.66	2	8.98	0.39	2
	2008	2340.67	1678.75	3	7.57	0.76	3	9839.77	2289.03	3	9.17	0.25	3
	2009	1961.67	908.60	3	7.51	0.45	3	14115.02	3803.33	3	9.53	0.26	3
	2010	1166.00	147.96	3	7.06	0.13	3	15595.23	1616.71	3	9.65	0.11	3
	2011	2020.50	672.84	4	7.57	0.33	4	15003.24	1753.48	4	9.61	0.12	4
SC	1998	1646.15	2382.98	33	6.17	1.91	33	.	.	0	.	.	0
	1999	1501.39	1956.31	33	6.21	1.85	33	.	.	0	.	.	0
	2000	1490.95	1961.48	19	6.27	1.75	19	.	.	0	.	.	0
	2001	1779.83	2216.75	18	6.59	1.69	18	4621.59	1062.77	16	8.41	0.27	16
	2002	2222.11	2600.56	18	6.55	2.19	18	5644.29	1634.80	17	8.61	0.25	17
	2003	2272.63	2844.52	16	6.54	2.06	16	7215.46	3583.34	16	8.82	0.33	16
	2004	1848.47	2666.79	15	6.22	2.07	15	10264.26	11306.69	12	9.00	0.57	12
	2005	2651.17	2905.95	12	6.91	1.86	12	7889.53	1919.36	9	8.95	0.26	9
	2006	2962.29	3299.72	14	7.04	1.71	14	9703.19	2700.44	12	9.15	0.27	12
	2007	2180.50	2545.26	14	6.80	2.15	14	9584.62	1983.42	13	9.15	0.21	13
	2008	2039.07	2559.10	15	6.73	1.78	15	12803.75	2583.14	14	9.44	0.21	14
	2009	1793.46	2073.91	13	6.53	1.84	13	14592.41	2403.82	11	9.58	0.16	11
	2010	2555.44	3614.53	9	6.70	2.10	9	15033.54	5085.04	9	9.57	0.31	9
	2011	2539.77	2831.04	13	6.61	2.49	13	13914.04	2890.61	13	9.52	0.24	13
SD	2002	831.13	1923.49	8	4.24	2.44	8	4442.12	1683.55	5	8.34	0.36	5
	2003	232.00	432.07	4	3.68	2.13	4	4868.34	1044.80	3	8.48	0.21	3
	2004	2099.40	3852.53	5	5.10	3.54	5	5432.60	1785.25	5	8.54	0.41	5
	2005	119.86	191.03	7	3.73	1.64	7	6187.08	1201.84	4	8.72	0.19	4
	2006	226.89	403.08	9	3.27	2.59	9	7275.17	3033.44	6	8.83	0.38	6
	2007	145.75	243.34	8	3.06	2.38	8	8160.88	2781.22	6	8.95	0.37	6
	2008	179.00	290.30	4	4.20	1.53	4	10415.01	4387.50	3	9.20	0.39	3
	2009	374.40	738.66	5	4.35	1.86	5	9488.79	4516.64	4	9.07	0.50	4
	2010	281.40	498.81	5	4.15	2.16	5	14013.05	3901.63	5	9.52	0.29	5
	2011	1116.33	2205.73	6	3.82	3.53	6	15859.38	3851.55	5	9.65	0.25	5
TN	1998	1833.24	3104.65	72	6.17	1.93	72	.	.	0	.	.	0
	1999	1727.65	3144.11	68	5.91	2.02	68	.	.	0	.	.	0
	2000	1470.77	2468.48	31	6.01	1.78	31	.	.	0	.	.	0
	2001	1438.00	2464.06	35	5.64	2.17	35	4562.21	1795.46	29	8.36	0.37	29
	2002	1845.00	3862.56	34	5.15	2.67	34	4310.55	1858.99	22	8.29	0.40	22
	2003	2207.50	4185.64	36	5.89	2.40	36	5381.90	1763.20	30	8.53	0.35	30
	2004	2265.75	4246.16	36	5.93	2.26	36	7562.90	6767.82	32	8.73	0.57	32
	2005	1990.11	3053.85	36	6.01	2.28	36	6936.59	2475.87	28	8.78	0.38	28
	2006	2795.21	3671.37	29	6.41	2.36	29	8230.80	3046.92	25	8.95	0.36	25
	2007	1455.17	2652.67	29	5.32	2.58	29	5339.41	2889.22	26	8.44	0.57	26
	2008	2638.70	4431.94	27	6.03	2.44	27	9429.04	2560.64	22	9.12	0.26	22
	2009	1651.36	2714.76	25	5.78	2.22	25	9848.75	3194.17	24	9.13	0.39	24
	2010	1537.18	2614.39	22	5.38	2.71	22	10252.25	3812.88	20	9.14	0.49	20
	2011	2447.79	4205.03	24	6.43	1.90	24	13518.88	5054.30	20	9.46	0.32	20
TX	2000	2280.67	3318.77	86	6.63	1.83	86	.	.	0	.	.	0
	2001	2366.51	2957.74	88	6.67	1.93	88	.	.	0	.	.	0
	2002	2147.48	2706.98	91	6.41	2.21	91	.	.	0	.	.	0
	2003	2335.74	3213.95	95	6.39	2.19	95	.	.	0	.	.	0
	2004	2265.84	3261.44	93	6.46	1.98	93	.	.	0	.	.	0
	2005	1772.31	2742.68	106	6.04	2.09	106	.	.	0	.	.	0
	2006	1778.82	2528.57	97	6.04	2.23	97	11686.68	10867.66	70	9.10	0.67	70
	2007	1753.61	2467.78	101	5.97	2.27	101	12129.51	11358.05	68	9.21	0.53	68
	2008	1813.89	2547.18	93	6.13	2.19	93	13974.49	12296.02	77	9.34	0.58	77
	2009	1787.28	2829.91	90	6.11	2.10	90	16376.05	13945.55	72	9.50	0.57	72
	2010	1653.68	2405.36	94	6.13	2.05	94	20025.22	16300.70	77	9.67	0.64	77
	2011	2068.35	3832.54	86	6.08	2.10	86	22936.73	17392.40	65	9.83	0.62	65
UT	1998	951.13	1272.27	16	5.66	1.82	16	.	.	0	.	.	0
	1999	1043.24	1710.57	17	5.59	1.93	17	.	.	0	.	.	0
	2000	303.07	341.56	14	5.03	1.27	14	.	.	0	.	.	0
	2001	1192.63	2499.33	16	5.49	1.91	16	3912.22	1205.23	14	8.23	0.31	14
	2002	1711.67	3005.40	15	5.70	2.27	15	4483.40	1303.96	13	8.37	0.27	13
	2003	2156.69	3032.01	13	6.24	2.06	13	5476.93	2136.16	13	8.55	0.35	13
	2004	1291.25	2351.85	12	5.45	2.13	12	5652.46	998.71	12	8.63	0.18	12
	2005	1149.21	1475.50	14	5.87	2.02	14	6114.30	1903.72	12	8.68	0.29	12
	2006	2051.31	3181.74	13	6.12	2.03	13	8116.45	2674.15	11	8.96	0.28	11
	2007	951.67	1642.12	12	5.76	1.68	12	8199.12	2194.26	11	8.98	0.29	11
	2008	1880.54	3130.25	13	6.00	2.09	13	12319.15	10752.92	12	9.24	0.53	12
	2009	1146.50	2418.00	8	5.08	2.18	8	19066.46	24818.49	8	9.47	0.79	8
	2010	2356.40	2982.92	10	6.76	1.83	10	12233.34	4199.51	10	9.36	0.34	10
	2011	2072.78	2315.16	9	6.69	1.83	9	11939.88	2741.62	9	9.36	0.23	9
VA	1999	2163.83	3145.65	47	6.86	1.51	47	.	.	0	.	.	0
	2000	2957.81	4416.64	21	6.99	1.51	21	.	.	0	.	.	0
	2001	2650.17	4247.87	24	6.65	2.07	24	5364.76	1758.76	21	8.55	0.28	21
	2002	2027.21	2361.63	19	7.01	1.15	19	5466.07	1717.03	18	8.57	0.27	18
	2003	3626.33	5260.93	18	7.37	1.31	18	6327.16	1961.95	17	8.71	0.28	17
	2004	3353.11	5117.73	18	7.08	1.69	18	7853.96	3415.77	16	8.90	0.36	16
	2006	3331.60	5168.00	20	6.70	2.24	20	9050.88	2449.50	19	9.08	0.26	19
	2007	3551.24	5334.38	17	6.82	2.07	17	10806.18	4125.97	15	9.22	0.37	15
	2008	2944.38	3510.67	16	6.77	2.06	16	11820.56	3187.60	16	9.35	0.26	16
	2009	3149.15	4461.06	20	6.88	2.18	20	15278.79	12479.85	20	9.50	0.44	20
	2010	3420.63	4320.27	19	6.91	2.25	19	14843.37	8297.78	19	9.52	0.39	19
	2011	2619.26	2948.99	23	6.98	1.61	23	15971.69	5915.47	22	9.62	0.33	22
VT	2001	821.25	680.87	4	6.38	0.99	4	5666.61	615.50	3	8.64	0.11	3
	2002	628.20	570.37	5	6.16	0.81	5	6101.65	1089.40	2	8.71	0.18	2
	2003	775.25	603.56	4	6.46	0.68	4	7457.04	904.78	4	8.91	0.12	4
	2004	1633.14	3181.02	7	6.43	1.24	7	8481.75	1315.27	5	9.04	0.16	5
	2005	1836.50	3429.40	6	6.45	1.42	6	10568.57	2445.73	5	9.25	0.22	5
	2006	1607.57	3159.69	7	6.33	1.37	7	13503.54	4372.78	7	9.47	0.30	7
	2007	2427.50	3864.77	4	6.88	1.46	4	12292.48	2834.93	4	9.40	0.23	4
	2008	686.00	432.75	2	6.42	0.68	2	16488.16	3945.03	2	9.70	0.24	2
	2009	360.50	236.88	2	5.77	0.71	2	16712.07	3012.68	2	9.72	0.18	2
	2010	2827.67	4280.13	3	6.89	1.81	3	19730.94	2612.98	3	9.88	0.13	3
	2011	2011.25	3598.76	4	5.98	2.17	4	25073.62	1485.64	4	10.13	0.06	4
WA	1998	1678.91	2740.89	22	6.05	2.01	22	.	.	0	.	.	0
	1999	1636.73	1930.03	22	6.37	1.84	22	.	.	0	.	.	0
	2000	2030.09	2969.30	23	6.60	1.63	23	.	.	0	.	.	0
	2001	1888.52	2971.38	23	6.57	1.57	23	4905.84	1289.30	18	8.46	0.27	18
	2002	1850.46	2308.31	24	6.20	2.23	24	6189.36	1986.10	16	8.69	0.26	16
	2003	2079.50	2411.59	20	6.39	2.09	20	7138.43	1931.02	16	8.84	0.26	16
	2004	1931.00	2234.20	22	6.55	1.77	22	8257.66	3086.56	17	8.97	0.30	17
	2005	1561.41	1940.25	22	6.49	1.54	22	13840.24	20265.70	17	9.18	0.68	17
	2006	1887.29	3003.83	24	6.38	1.90	24	9767.47	3330.06	19	9.14	0.28	19
	2007	2212.80	3195.13	20	6.37	2.15	20	13778.54	13718.12	18	9.33	0.53	18
	2008	1838.39	2499.92	18	6.25	2.26	18	12219.06	3747.48	18	9.37	0.29	18
	2009	1748.53	2023.22	17	6.37	1.89	17	15604.95	6515.85	17	9.58	0.38	17
	2010	1930.28	2773.72	18	6.43	1.67	18	19868.53	8770.69	17	9.82	0.40	17
	2011	1671.89	2321.80	19	6.29	1.71	19	18375.82	8458.34	17	9.74	0.39	17
WI	1998	1625.94	2337.00	66	6.45	1.51	66	.	.	0	.	.	0
	1999	1492.06	2452.53	65	6.13	1.80	65	.	.	0	.	.	0
	2000	1558.05	2495.54	66	6.23	1.66	66	.	.	0	.	.	0
	2001	1477.54	2870.39	35	6.04	1.77	35	4836.80	1495.99	24	8.44	0.28	24
	2002	1438.72	1895.53	29	6.35	1.57	29	6162.16	1674.11	23	8.70	0.25	23
	2003	1226.64	1772.33	28	5.92	1.73	28	6551.88	1445.91	18	8.76	0.23	18
	2004	1504.37	2333.54	27	6.22	1.58	27	7589.25	1888.95	21	8.90	0.27	21
	2005	1478.83	2072.28	30	6.24	1.63	30	8909.31	2088.56	26	9.07	0.23	26
	2006	1660.79	3368.63	29	5.83	2.07	29	10397.91	1885.77	24	9.23	0.17	24
	2007	1297.37	2005.02	27	5.99	1.80	27	12846.44	9489.97	25	9.35	0.39	25
	2008	1593.83	2328.41	30	6.07	2.07	30	12701.90	2608.61	28	9.43	0.22	28
	2009	1259.47	2216.66	30	5.74	2.12	30	18466.41	15143.07	29	9.67	0.48	29
	2010	1736.61	2211.16	28	6.11	2.21	28	17365.88	10329.55	27	9.69	0.33	27
	2011	1432.87	2180.68	30	5.91	2.19	30	17795.42	5877.10	27	9.73	0.38	27
WV	2000	472.63	557.48	19	4.94	2.21	19	.	.	0	.	.	0
	2001	248.80	278.53	15	4.51	1.88	15	3888.09	534.79	5	8.26	0.13	5
	2002	1351.47	3077.94	19	5.29	2.30	19	4409.61	2031.18	8	8.27	0.57	8
	2003	1049.50	1569.87	18	5.31	2.37	18	5942.96	2070.32	16	8.63	0.36	16
	2004	814.67	1118.55	15	5.64	1.77	15	5359.41	1357.20	6	8.56	0.25	6
	2005	2531.00	3497.98	14	6.77	2.06	14	7780.89	2124.96	9	8.92	0.29	9
	2006	823.38	1262.49	13	5.26	2.11	13	7300.30	1453.31	6	8.88	0.20	6
	2007	1574.67	2429.76	15	5.42	2.68	15	9333.81	3269.12	8	9.09	0.32	8
	2008	1678.00	2495.48	13	5.05	3.14	13	10792.28	5094.92	11	9.17	0.52	11
	2009	919.25	1309.24	12	5.92	1.59	12	10234.95	1996.01	11	9.22	0.19	11
	2010	1210.15	1406.11	13	6.14	1.83	13	14794.36	10101.22	11	9.48	0.45	11
	2011	2780.43	4238.53	7	6.30	2.34	7	19117.61	11847.47	7	9.74	0.48	7
WY	2007	430.00	270.70	4	5.90	0.67	4	12490.09	4404.88	4	9.39	0.35	4
	2008	257.00	318.24	6	4.54	1.86	6	13683.53	3694.38	4	9.50	0.25	4
	2009	414.00	355.24	7	5.56	1.16	7	14386.57	4346.47	7	9.54	0.29	7
	2010	319.14	300.32	7	5.17	1.38	7	13824.34	1395.73	7	9.53	0.11	7
	2011	405.00	254.13	6	5.58	1.35	6	17362.34	5164.73	6	9.72	0.33	6

**Table 8:** Descriptive for the main outcomes in outpatient file (SASD)

Hospital state	Calendar year	Total number of surgical procedures			Log of total number of surgical procedures		
		Mean	Std	N	Mean	Std	N
CA	2007	3515.62	3625.26	853	7.57	1.32	852
	2008	3432.36	3886.85	831	7.48	1.38	831
	2009	4068.27	4485.46	584	7.73	1.24	584
	2010	4377.43	4874.96	497	7.78	1.27	497
	2011	4527.95	5071.43	458	7.77	1.37	458
CO	2008	4870.29	5532.98	78	7.42	2.09	78
	2009	5103.38	5509.80	74	7.67	1.74	74
	2010	5498.03	5641.74	73	7.80	1.62	73
	2011	5576.49	5952.14	78	7.77	1.66	78
	2012	5473.14	5843.59	78	7.65	1.89	78
FL	2007	5410.50	5283.45	572	8.19	1.03	572
	2008	5337.35	5276.53	587	8.17	1.05	587
	2009	5223.05	4927.84	588	8.17	1.01	588
	2010	5109.85	4912.04	590	8.13	1.04	590
	2011	4832.48	4472.26	605	8.08	1.06	605
	2012	4834.20	4207.72	599	8.13	0.95	599
	2013	4792.27	4237.82	605	8.09	1.05	605
KY	2007	7385.13	8979.66	104	8.13	1.42	104
	2008	13539.70	16737.80	105	8.86	1.21	105
	2009	13416.91	17141.52	131	8.83	1.22	131
	2010	11449.95	14604.36	150	8.67	1.24	150
	2011	10718.50	14358.56	164	8.53	1.39	164
	2012	10301.86	14306.77	177	8.38	1.52	177
	2013	14892.33	20248.58	206	8.69	1.58	206
MD	1998	6807.10	5681.28	52	8.49	0.90	52
	1999	6884.13	5819.98	52	8.47	0.96	52
	2000	7560.06	6166.07	49	8.64	0.82	49
	2001	8000.85	6432.89	48	8.66	0.93	48
	2002	8250.75	6402.70	48	8.74	0.80	48
	2003	17713.54	22895.29	48	9.29	0.96	48
	2004	18459.33	23312.07	48	9.39	0.85	48
	2005	19235.23	23951.71	48	9.43	0.88	48
	2006	20425.46	26811.17	48	9.48	0.86	48
	2007	38515.06	44829.21	52	9.93	1.45	52
	2008	64059.83	71433.03	52	10.52	1.35	52
	2009	65421.62	84632.03	52	10.51	1.35	52
	2010	65719.71	86014.19	51	10.51	1.34	51
	2011	64722.55	89320.17	53	10.26	1.83	53
	2012	64992.65	93877.46	54	10.13	2.11	54
NJ	2008	5504.35	4211.93	79	8.13	1.33	79
	2009	6039.51	4190.19	75	8.28	1.32	75
	2010	6359.12	4434.84	74	8.40	1.12	74
	2011	6489.85	4579.18	74	8.39	1.23	74
	2012	6287.65	4574.22	75	8.26	1.53	75
	2013	6199.96	4668.46	75	8.22	1.48	75

## Footnotes

1 In NIS, the total number of all surgeries was the sum of all hospitalizations with surgical DRG in a facility (excluding records with patients age younger than 1); In SASD, it was the total number of visits in the facility.

2 We used the size classification defined by HCUP, for which specific bed-size thresholds for size categories vary across Census regions, and by urban/rural and teaching status (https://www.hcup-us.ahrq.gov/db/vars/hosp_bedsize/nisnote.jsp).

3 These facility level variables were almost fixed over the sample time period. Dropping the facility variables from the facility fixed-effects model does not change model results.

4 The market area definition recommended by HCUP was used (see *HCUP Hospital Market Structure File: 2009 Central Distributor SID, NIS, and KID User Guide* [https://www.hcup-us.ahrq.gov/toolssoftware/hms/HMSUserGuide2009.pdf].) Years with missing HHI values were imputed using a time trend.

5 The source for these data is county-level data from the Area Resource File (ARF).

6 Estimated as ${\upbeta}^{*}=\left[ \exp \left[\widehat{\upbeta}\hbox{-} \frac{1}{2}\operatorname{var}\left(\widehat{\upbeta}\right)\right]-1\right]$. See Kennedy [12].
